# Perinephric Abscess Extending to the Psoas Muscle and Causing a Nephro-Cutaneous Fistula

**DOI:** 10.7759/cureus.35662

**Published:** 2023-03-01

**Authors:** Mulham Alom, Victoria Bengualid

**Affiliations:** 1 Internal Medicine, St. Barnabas Hospital Health System, Bronx, USA; 2 Infectious Diseases, St. Barnabas Hospital Health System, Bronx, USA

**Keywords:** perinephric abscess, bacteroides species, citrobacter koseri, secondary psoas abscess, nephro-cutaneous fistula

## Abstract

We present a case of a 76-year-old male with dementia transferred from a nursing home with a fever and an abscess on his back. Workup revealed an extensive perinephric abscess, which extended to his psoas muscle, with an additional fistula to his back where the abscess was noted. The extent and tracking of the perinephric abscess were unusual as well as the organisms isolated, *Citrobacter koseri* and *Bacteroides *species.

## Introduction

Perinephric abscesses are rare, with an incidence of one per 10,000 hospital admissions [1]. They can occur as a complication of urological procedures and are more common on the right side [1]. Perinephric abscesses most commonly occur as a complication of renal infections such as chronic pyelonephritis, rupture of an intrarenal abscess into the perinephric space, and xanthogranulomatous pyelonephritis [1]. Perinephric abscesses are diagnosed by imaging as signs and symptoms are nonspecific. Early diagnosis is important to avoid complications and increased morbidity. Most cases of perinephric abscesses are caused by gram-negative organisms such as *Escherichia coli* and *Proteus *spp., which when combined are responsible for 50% of the infections [1]. Gram-negative infections are presumed due to infections ascending from the ureter. Infections caused by *Staphylococcus aureus* usually occur via blood seeding of the kidneys or the perinephric space [2]. The most important risk factors for developing perinephric abscess are nephrolithiasis (in 50% of the cases), diabetes mellitus, and urological procedures [3].

## Case presentation

A 76-year-old male was transferred from the nursing home on account of a fever and an abscess on his back. He had a history of advanced dementia, atrial fibrillation, diabetes mellitus, hypertension, depression, and seizures. In the emergency room, on physical exam, he had a temperature of 38.6 °C, a heart rate of 120 beats/minute, and a blood pressure of 106/56 mmHg. The respiratory rate was 18 breaths per minute. He had an area of induration with redness on his mid-back, but no drainage was noted. Laboratory results were notable for elevated lactic acid levels, hyponatremia, elevated creatinine, and leukocytosis (Table 1). He was given intravenous (IV) fluids. Blood cultures were drawn. Urinalysis was significant for pyuria 182 white blood cells per high power field (WBCs/HPF). Chest X-ray was normal. He was started on empiric antibiotic coverage (vancomycin and cefepime) and admitted under the impression of sepsis.

**Table 1:** Laboratory values. BUN, blood urea nitrogen; WBC, white blood cell

Laboratory value	Admission day 1	Admission day 3	Reference range
Lactic acid (mm/L)	4.7	1.8	<2
Sodium (mEq/L)	129	137	135-145
Creatinine (mg/dL)	1.5	1.8	0.6-1.1
BUN (mg/dL)	26	25	8-23
WBC (1,000 mm^-3^)	13.1	10.6	4-12

**Figure 1:**
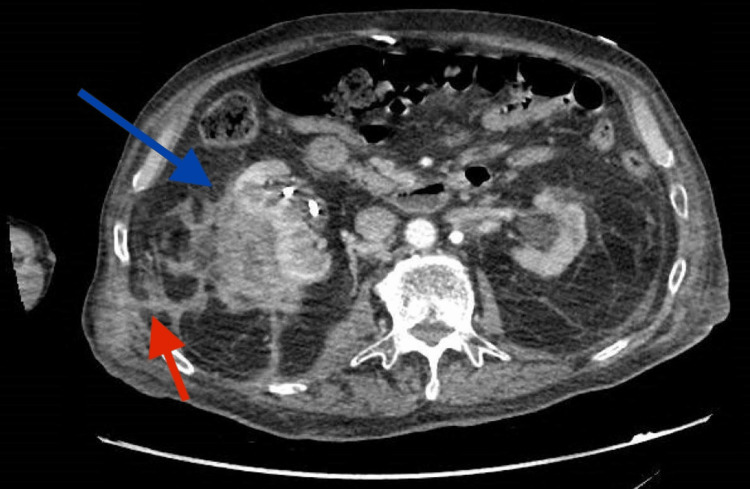
Axial view of the perinephric abscess (blue arrow), with the red arrow pointing to the fistula tract exiting in the patient’s back, forming an abscess.

**Figure 2:**
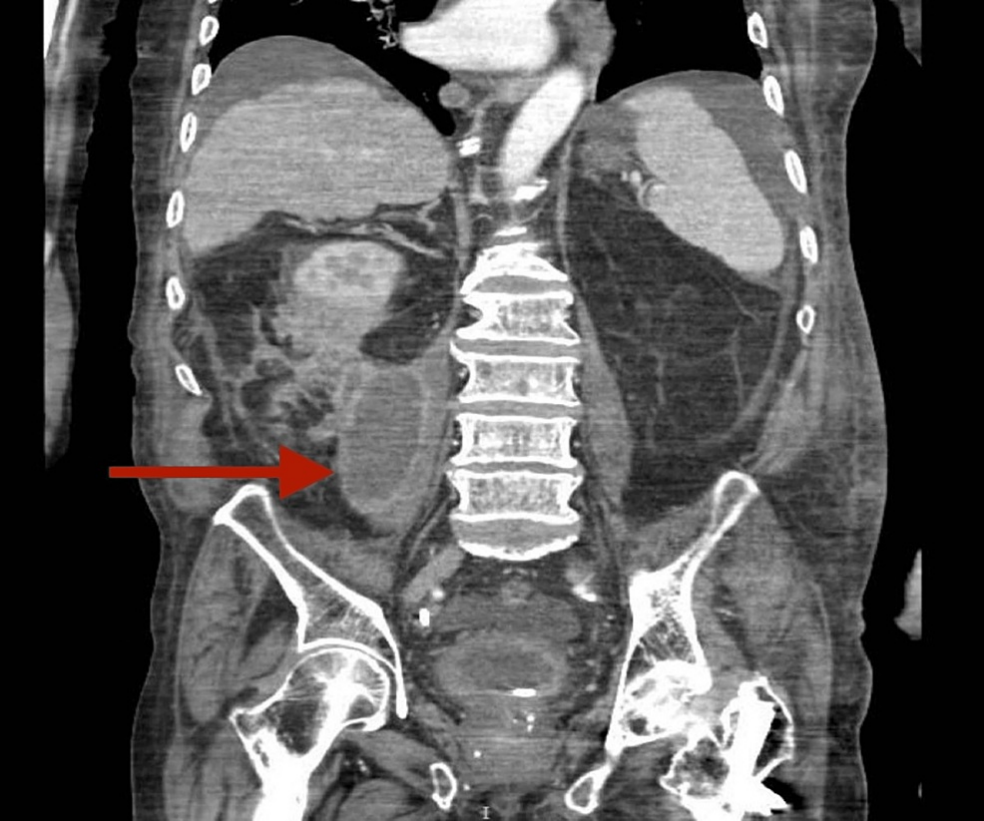
Coronal view showing perinephric abscess extending to the psoas muscle forming a psoas abscess (red arrow).

**Figure 3:**
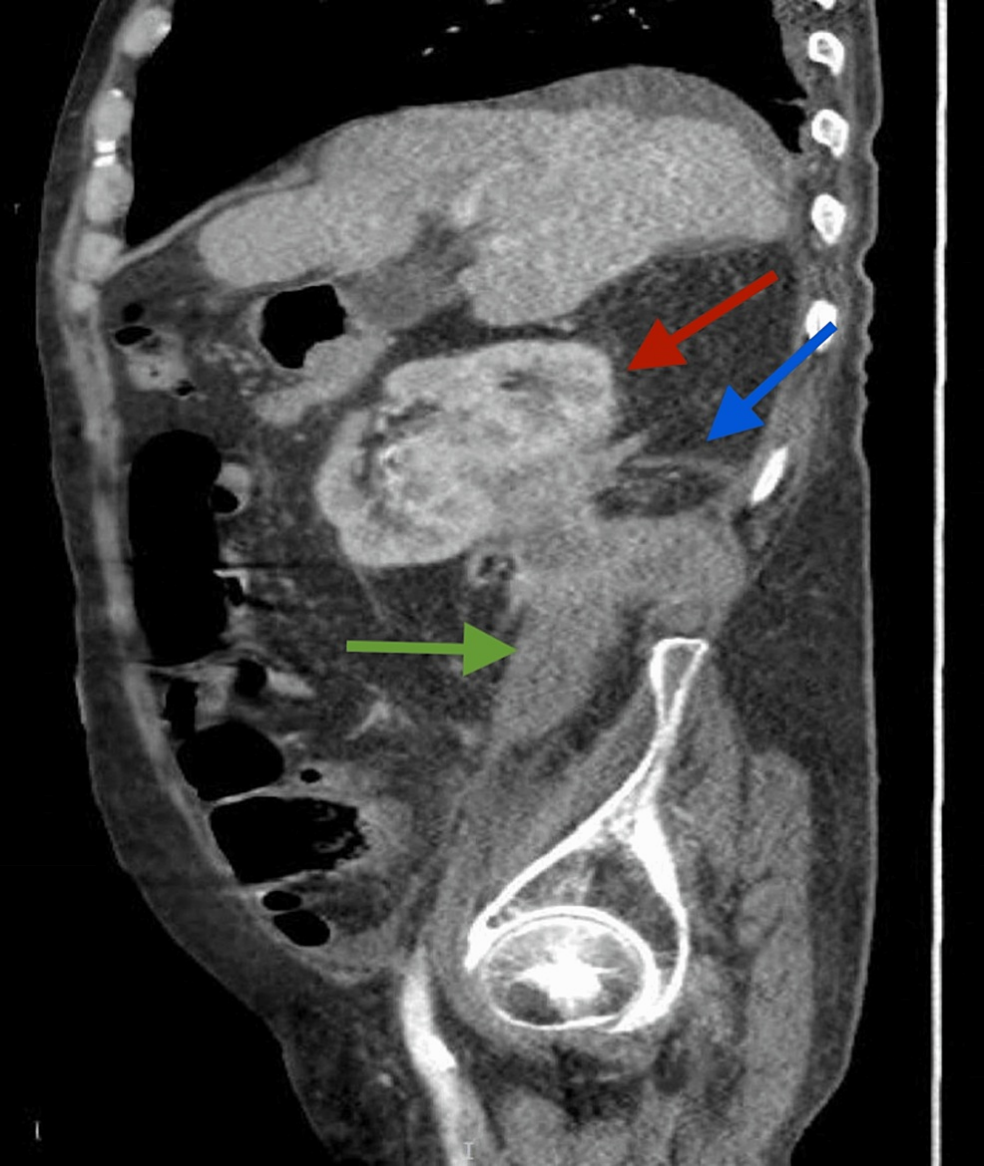
Computed tomography scan showing extensive perinephric abscess (red arrow), psoas abscess (green arrow), and perinephric cutaneous fistula to the back (blue arrow).

A contrast-enhanced computed tomography (CT) scan of the abdomen and pelvis was obtained to further evaluate the abscess on his back. It showed a right ureteral stent and an obstructing stone of the left ureter with hydronephrosis. However, the most striking feature was a right perinephric abscess that extended into the psoas muscle. The perinephric abscess was tracked dorsally with an exit site in the back where the patient had the findings of an abscess on physical exam (Figures 1-3). Management of the left kidney stone was overseen by the urologist who felt that he would be able to pass the stone with fluids and conservative management.

The patient underwent an ultrasound-guided percutaneous psoas abscess drainage by interventional radiology. During the procedure, the saline used to wash out the cavity also drained from the perinephric cutaneous fistula, confirming the connection between the psoas abscess, the perinephric abscess, and the perinephric cutaneous fistula/abscess previously noted on the patient’s back. A drain was left in place. The perinephric abscess grew *Citrobacter koseri* (which also grew in urine cultures) and *Bacteroides fragilis*. Blood cultures grew *Bacteroides* species. Antibiotic coverage was narrowed to ceftriaxone and metronidazole. The patient improved clinically, and a repeat CT scan one week later showed a resolution of the prior noted abscess. The drain was removed, and the patient completed a two-week course of antibiotics.

## Discussion

The diagnosis of a perinephric abscess is difficult to make by history or physical exam as it presents with nonspecific findings. This patient's presentation was further obscured by his dementia. Our patient did present with a fever, which is present in 90% of the cases [4]. Other presenting signs and symptoms include costovertebral tenderness (88% of cases) and lumbar pain (77% of cases) [3]. Laboratory findings include leukocytosis and pyuria, both of which were present in our patient. The patient did have risk factors for developing a perinephric abscess, which included his diabetes and previous urologic procedures.

The diagnosis was made by a CT scan, which is the gold standard and can help differentiate other entities [1]. Prompt drainage, usually by interventional radiology, and antibiotic therapy improve mortality [5]. Optimally drainage catheter should be left in place until the resolution of the abscess on repeat imaging or if the patient is showing signs of improvement clinically [1]. In our patient, CT imaging showed extensive abscess formation in the psoas muscle and tracking from the perinephric collection to the skin causing an abscess. As our patient was not verbal or able to express discomfort, it remains unclear how long he had this ongoing process.

Our patient grew *C. koseri *and *Bacteroides *species. *C. koseri* is an opportunistic gram-negative bacteria that have been isolated with increased frequency from the urine of hospitalized patients. Risk factors for this pathogen include immunocompromised states such as his diabetes, the presence of an indwelling Foley catheter (which he had in the past), and a history of genitourinary instrumentation or obstruction [6] (our patient had a left kidney stent). Renal or perinephric abscess formation with *C. koseri* is unusual, with five previous cases reported in the literature [7]. Treatment includes antibiotics and drainage of abscesses of 5 cm or greater.

Our patient also had blood and urine cultures positive for *Bacteroides *species. There have not been any reported cases of co-infection with these two pathogens. However, a study conducted in 1988 isolated *Bacteroides *spp. from 17% of perinephric abscesses [8]. Another study published in 2004 described the role of anaerobes in genitourinary suppurative infections [9]; in this study, *Bacteroides *spp. were isolated in combination with an aerobic pathogen. They noted that recurrent urinary tract infections with an obstructive component, especially in diabetic patients, often progress to chronic renal disease that can progress to renal and perinephric abscesses [9].

## Conclusions

We describe a case of a perinephric abscess causing a fistula to the patient’s back and extending to his psoas muscle, causing a large psoas abscess. Perinephric abscesses are difficult to diagnose as symptoms can be nonspecific, especially difficult in patients with dementia. Early drainage and antibiotics are the cornerstones of treatment. Cultures from the abscess grew *C. koseri* and *B. fragilis*. *C. koseri* is an uncommon pathogen causing urinary tract infections. It has only been reported in five previous cases of perinephric abscesses, and the combination with *B. fragilis* has not been previously described.
